# Investigation on flavonoid composition and anti free radical potential of *Sida cordata*

**DOI:** 10.1186/1472-6882-13-276

**Published:** 2013-10-22

**Authors:** Naseer Ali Shah, Muhammad Rashid Khan, Bushra Ahmad, Farah Noureen, Umbreen Rashid, Rahmat Ali Khan

**Affiliations:** 1 Department of Biochemistry, Faculty of Biological Sciences, Quaid-i-Azam University, Islamabad 45320, Pakistan; 2 Deparment of Biotechnology, Faculty of Biological Sciences, University of Science and Technology Bannu, Khyber Pakhtunkhwa, Pakistan

**Keywords:** *Sida cordata*, Phytochemistry, Antioxidant assays, CCl_4_, Liver toxicity

## Abstract

**Background:**

*Sida cordata*, a member of Family Malvaceae is used in folk medicine for various ailments including liver diseases. In this study we investigated, its flavonoid constituents, *in vitro* antioxidant potential against different free radicals and hepatoprotection against carbon tetrachloride (CCl_4_)-induced liver damage in rat.

**Methods:**

Dried powder of *S. cordata* whole plant was extracted with methanol and the resultant (SCME) obtained was fractionated with escalating polarity to obtain *n*-hexane fraction (SCHE), ethyl acetate fraction (SCEE), *n*-butanol fraction (SCBE) and the remaining soluble portion as aqueous fraction (SCAE). Diverse *in vitro* antioxidants assays such as DPPH, H_2_O_2_, •OH, ABTS, β-carotene bleaching assay, superoxide radical, lipid peroxidation, reducing power, and total antioxidant capacity were studied to assess scavenging potential of methanol extract and its derived fractions. On account of marked scavenging activity SCEE was selected to investigate the hepatoprotective potential against CCl_4_ induced toxicity in Sprague–Dawley male rats by assessing the level of serum markers (alkaline phosphatase, alanine transaminase, aspartate transaminase, lactate dehydrogenase, bilirubin, and γ-glutamyltransferase) and of liver antioxidant enzymes such as catalase (CAT), superoxide dismutase (SOD), peroxidase (POD), glutathione-S-transfers (GST), glutathione reductase (GSR), glutathione peroxidase (GSH-Px), and reduced glutathione (GSH) and lipid peroxidation (TBARS). Histology of the liver was performed to study alteration in histoarchitecture. Existence of active flavonoids was established by thin layer chromatographic studies.

**Results:**

Considerable amount of flavonoid and phenolic contents were recorded in the methanol extract and its derived fractions. Although the extract and all its derived fractions exhibited good antioxidant activities however, the most distinguished scavenging potential was observed for SCEE. Treatment of SCEE decreased the elevated level of serum marker enzymes induced with CCl_4_ administration whereas increased the activity of hepatic antioxidant enzymes (CAT, SOD, POD, GST, GSR and GSH-Px). Hepatic concentration of GSH was increased while lipid peroxidation was decreased with SCEE administration in CCl_4_ intoxicated rats. Presence of apigenin with some unknown compounds was observed in SCEE by using thin layer chromatography.

**Conclusions:**

These results revealed the presence of some bioactive compound in the ethyl acetate fraction, confirming the utility of *S. cordata* against liver diseases in folk medicine.

## Background

Free radicals are classified having an unpaired electron. They belong to extremely reactive species, generated continuously in cells either as by-products of metabolism or by leakage from mitochondrial respiration [1]. In the previous decade, there has been a mounting interest in the medical implications of free radicals. Free radicals such as superoxide radical (O_2_^-^), hydroxyl radical (•OH), hydrogen peroxide (H_2_O_2_) and lipid peroxides (LOOH) are becoming of enormous concern in human diseases. They are recognized for DNA damage, lipid peroxidation and protein breakdown along with role in the pathogenesis of many clinical disorders such as inflammatory diseases, cardiac diseases, asthma, Alzheimer’s, Parkinson’s and aging [2,3]. Radicals such as O_2_^-^, •OH, and H_2_O_2_ etc. arbitrate components of the inflammatory response, with production of cyclic nucleotides, migratory factors and eicosanoids. Superoxide radicals intensify the inflammatory process by adhesion of polymorphonuclear leukocytes to the endothelium, increasing vascular permeability and stimulation of platelet aggregation [4]. Free radicals and reactive oxygen species are also the major cause of introducing genetic mutation leading to different sorts of cancer. Evidence is growing for a role of dietary phytochemicals, including ascorbic acid, flavonoids, carotenoids and tocopherol as an antioxidants in the maintenance of health and resistance to diseases [5].

Natural products such as flavonoids and phenolics have been observed to be efficient free radical scavengers and lipid peroxidation inhibitors [6,7]. Several synthetic based antioxidant compounds have proved to be toxic and/or mutation inducer, resulting in attention of many researchers to quest for natural antioxidants.

*S. cordata*, member of the Malvaceae Family, is a trailing way side herb found frequently growing in shady places. It is cosmopolitan in Pakistan, India and other tropical countries and is mostly distributed in clearing in the forest and as weeds in the overgrown grass of gardens and public parks [8]. It is known as Farid buti, Rajbala, Bhumibala and Shaktibala in India [9] and Simak in Pakistan [10]. It is extensively used for therapeutic purposes in the codified Indian systems of medicine namely Siddha and Ayurveda. Its roots are used as diuretic, astringent, stomachic, febrifuge and demulcent and seeds are applied as laxative, aphrodisiac and demulcent; recommended in cystitis, colic gonorrhea, tenseness, and piles [10]. It has a potential for strengthening and glowing of the body [9]. In folk medicine, female uses it as soup in the last days of pregnancy to reduce pain of labor and reducing the period. It also leads to decrease the period of post parturition bleeding [11]. The abortifacient effect of alcoholic extract is also reported in pregnant rats [8].

The whole plant material is used for chronic hepatic diseases [12]. Its hepatoprotective effects have been investigated in an *in vitro* study [13]. Mistry et al. [14] reported that crude ethanol extract of the leaves of *S. cordata* is hepatoprotective against CCl_4_ induced toxicity in rat. However, a systematic approach is required to determine the main fraction involved in the antioxidant potential of this plant. The present study was under taken to evaluate the methanol extract and its derived fractions through various *in vitro* anti free radical assays with subsequent use of the desired fraction to investigate its antioxidant capacity against CCl_4_ induced hepatic toxicity in animal model. Methanol extract and all its derived fractions were additionally subjected to the total flavonoid content, total phenolic content and to establish the existence of various active flavonoid constituents by thin layer chromatography.

## Methods

### Plant collection and preparation of extract

The whole plant was collected from the campus of Quaid-i-Azam University, Islamabad, Pakistan and recognized by their local names and then confirmed by Prof. Dr. Mir Ajab Khan, Department of Plant Sciences, Quaid-i-Azam University, Islamabad and Dr. Muhammad Zafar, Curator, Herbarium, Quaid-i-Azam University, Islamabad. Voucher specimen with accession No. 27824 was deposited at the Herbarium, Quaid-i-Azam University, Islamabad.

Shade dried 4 kg powder of *S. cordata* whole plant was extracted twice for 72 h in 8 L of methanol and filtered through Whatmann filter paper # 45, and the filtrate was concentrated through rotary vacuum evaporator at reduced pressure to get methanol extract (SCME). To sort the extract in increasing order of polarity it was suspended in distilled water (6 g/250 ml) and passed through different solvents (250 ml each) in the order of n-hexane (SCHE)→ethyl acetate (SCEE)→n-butanol (SCBE) to get different fractions by using separating funnel. The soluble residue was termed aqueous fraction (SCAE). All the fractions were stored at 4°C until further use.

### Phytochemical analysis

#### Total phenolic content

Spectrophotometric method [15] was used for determination of total phenolic content. In short, 1 ml of the extract and its derived fractions (1 mg/ml) were mixed with 1 ml of Folin-Ciocalteu’s reagent followed by Na_2_CO_3_ (7%, 10 ml) after 5 min. Mixture was thoroughly mixed with 13 ml of deionized distilled water and incubated at 23°C in the dark. After 90 min, absorbance was recorded at 750 nm. Total phenolic content was calculated from calibration curve of gallic acid serial dilutions. Estimation of phenolic compounds was recorded in triplicate and expressed as mg of gallic acid equivalents (GAE) per g of dried extract.

#### Total flavonoid content

In test tubes, samples (0.3 ml) of *S. cordata* were thoroughly mixed with 30% methanol, 0.5M NaNO_2_ (0.15 ml) and 0.3 M AlCl_3_.6H_2_O (0.15 ml) followed by addition of 1 ml NaOH (IM) after 5 min. Absorbance was measured at 506 nm against the reagent blank. Total flavonoid content was estimated by using a calibration curve of rutin and expressed as mg rutin equivalents per g of dried extract [16].

#### Thin layer chromatography

Extract and all fractions of *S. cordata* were dissolved (60 mg/ml) separately in HPLC grade methanol [17]. Silica gel TLC plates were cut into 20 × 20 cm sections. Each section was marked at 1 cm from one side. A volume of 10 μl of each sample and standard compounds such as myricetin, rutin, apigenin, kaempherol, catechin, quercetin, tannic acid, ascorbic acid, salicylic acid and caffeic acid were spotted by using a capillary tube on the line marked at one corner of the plate. Plates were allowed to develop after 20 min of vapor saturation in 120 ml of mobile phase; *n*-butanol, acetic acid and water (4:1:5). Plates were moved out when the mobile phase reached 1 cm below at the upper end. Solvent front was marked with lead pencil, air dried. The plates were dipped in a solution of 1% ethanolic 2-aminoethyle diphenyl borinate followed by a 5% ethanolic solution of polyethylene glycol-400. Phenolics and flavonoids were identified through its attributed colors under UV at 365 and 255 nm. RF values were calculated as:

$$\begin{array}{l} \text{RF}=\text{Distance}\;\text{covered}\;\text{by}\;\text{spot}/\text{Distance}\;\text{covered}\\ \;\text{by}\;\text{mobile}\;\text{phase} \end{array}$$

### Antioxidant assays

#### Samples preparation

*S. cordata* extract, fractions and positive standards (ascorbic acid, butylated hydroxytoluene, catechin and gallic acid) 200 μg were dissolved in 1 ml analytical methanol. These solutions were further serially diluted to 100 μg/ml and 50 μg/ml. In all the different antioxidant assays, same dilutions of sample and standards were used; while standard altered as per assay requirement.

#### DPPH radical scavenging assay

The DPPH (1, 1-diphenyl-2-picryl-hydrazyl) assay was performed according to the protocol of Sirajuddin et al. [18]. DPPH (24 mg) was dissolved in 100 ml methanol (stock solution). The solution was stored at 20°C until required. A working solution was made by diluting DPPH stock solution by methanol until the absorbance of 0.98± 0.02 was obtained at 517 nm. Working DPPH solution (0.9 ml) was added to 100 μl of various concentrations of test samples and incubated for 60 min in the dark at room temperature after being shaken well. Subsequently, the absorbance of the test samples was recorded at 517 nm. Ascorbic acid was used as standard.

Scavenging activity was calculated using the following equation

$$\begin{array}{l} \text{DPPH}\;\text{radical}\;\text{Scavering}\;\text{effect}\;\left(\%\right)\\ \;=\left[\left(\text{control}‒\text{sample}\right)/\left(\text{control}\right)\right]\times 100 \end{array}$$

#### Hydrogen peroxide scavenging assay

The method of Bokhari et al. [19] was followed to investigate hydrogen peroxide scavenging capacity of samples. Hydrogen peroxide (2 mM) solution was prepared in phosphate buffer (50 mM, pH 7.4). Samples (100 μl) were pipetted into eppendorfs and their volume made up to 400 μl with 50 mM phosphate buffer (pH 7.4). H_2_O_2_ solution (600 μl) was added and absorbance at 230 nm was taken 10 min after vortexing the eppendorfs. Percent scavenging activity was determined by following formula;

$$\begin{array}{l} H_{2}O_{2}\%\text{scavenging}\;\text{activity}\\ \;=\left(1‒\text{absorbance}\;\text{of}\;\text{sample}/\text{absorbance}\;\text{of}\;\text{control}\right)\times 100 \end{array}$$

Ascorbic acid served as standard.

#### Hydroxyl radical scavenging assay

The antioxidant activity was evaluated by method reported by Halliwell and Gutteridge [20]. The reaction mixture comprised of 2-deoxyribose (2.8 mM, 500 μl) in 50 mM of phosphate buffer, 100 μl of 0.2 M hydrogen peroxide solution, 200 μl of 0.1 M ferric chloride, 0.1M EDTA and 100 μl of test sample. The reaction was initiated by the addition of 100 μl of ascorbate (0.3 M). The mixture was incubated at 37°C for 60 min. TCA (2.8% w/v, 1 ml) and 1 ml of thiobarbituric acid (TBA) solution in 50 mM of sodium hydroxide (1%; w/v) was added. This reaction mixture was heated for 15 min in boiling water bath and then allowed to cool. Absorbance was recorded at 532 nm.

$$\begin{array}{l} \text{Hydroxyl}\;\text{scavenging}\;\text{activity}\;\left(\%\right)\\ \;=1‒\left(\text{Asorbance}\;\text{of}\;\text{sample}/\text{Absorbance}\;\text{of}\;\text{control}\times 100\right) \end{array}$$

#### ABTS radical cation scavenging activity

Re et al. [21] methodology with slight modification was followed for ABTS (2, 2 azobis, 3-ethylbenzothiozoline-6-sulphonic acid) radical cation scavenging activity. ABTS (7 mM) solution was reacted with 2.45 mM potassium persulfate and kept overnight in dark for generation of dark colored ABTS radicals. For the assay, the solution was diluted with 50% ethanol for an initial absorbance of 0.7 at 745 nm. Activity was determined by adding 100 μl sample of different dilution with 1 ml of ABTS solution in glass cuvette. Decrease in absorbance was measured after one min and 6 min of mixing. The difference was calculated and compared with control. Percent inhibition was calculated by following formula;

$$\begin{array}{l} \text{ABTS}\;\text{scavenging}\;\text{effect}\;\left(\%\right)\\ \;=\left[\left(\text{control}\;\text{absorbance}‒\text{sample}\;\text{absorbance}\right)/\right)\\ \;\left(\left(\text{control}\;\text{absorbance}\right)\right]\times 100\; \end{array}$$

#### Anti lipid peroxidation assay

This assay was performed as illustrated by Dorman et al. [22]. An aliquot of egg yolk (10%, w/v) was prepared in KCl (1.15%, w/v). The yolk was homogenized for 30 sec and subsequently subjected to ultrasonication for 5 min. Each sample (100 μl) at varying concentrations (200, 100, 50 μg/ml in methanol) and 500 μl of yolk homogenate were pipetted into eppendorfs and volume was made up to 1 ml with distilled water. It was mixed with 1.5 ml of acetic acid (20%, pH 3.5) and TBA (0.8%, w/v) in sodium dodecyl sulphate (1.1%, w/v). The reaction mixture was vortexed and incubated for 60 min in water bath. *n-*Butanol was added after cooling at room temperature, stirred and then centrifuged for 10 min at 3000 rpm. Butylated hydroxytoluene served as standard. The absorbance at 532 nm of supernatant was recorded.

The percent anti lipid peroxidation was determined by formula (1 ‒ S/C) × 100

Where

C = Absorbance of control and S = Absorbance of test sample

#### β-Carotene bleaching assay

Elzaawely et al. [23] modified method was used for β-carotene bleaching assay. β-carotene (2 mg) was dissolved in 10 ml of chloroform and blended with 20 mg of linoleic acid and 200 mg of Tween 80 followed by removal of chloroform under nitrogen with subsequent addition of 50 ml of distilled water with vigorous shacking to prepare β-carotene linoleate emulsion. An aliquot of each sample (50 μl) was mixed with 1ml of the emulsion, vortexed and absorbance was determined at 470 nm immediately against the blank solution. Capped tube was then kept in a water bath at 45°C for 2 h and the difference between the initial readings is calculated by measuring the reading after 2 h. β-Carotene bleaching inhibition was estimated by the following equation:

$$\text{Bleaching}\;\text{inhibition}\;\left(\%\right)=\left(A_{0t}‒A_{120t}/A_{0C}‒A_{120C}\right)\times 100$$

#### Superoxide anion radical scavenging assay

Riboflavin light NBT system assay was followed for superoxide radical scavenging activity [24]. The reaction mixture contained 0.5 ml of phosphate buffer (50 mM, pH 7.6), 0.3 ml riboflavin (50 mM), 0.25 ml PMS (20 mM), and 0.1 ml NBT (0.5 mM), prior to the addition of 1 ml sample in methanol. Florescent lamp was used for starting the reaction. Absorbance was recorded at 560 nm after incubation of 20 min under light. The percent inhibition of superoxide anion generation was calculated using the following formula:

$$\begin{array}{l} \text{Percent}\;\text{scavenging}\;\text{activity}\;\left(\%\right)\\ \;=\left(1‒\text{Absorbance}\;\mathrm{of}\;\text{sample}/\text{Absorbance}\;\text{of}\;\text{control}\right)\times 100 \end{array}$$

#### Reducing power activity assay

Reducing power of test samples was determined following modified protocol reported by Oyaizu [25]. A volume of 100 μl of various concentrations (50, 100 and 200 μg/ml) of test samples, 100 μl of phosphate buffer (0.2 M, pH 6.6) and 100 μl of potassium ferricyanide (10 mg/ml) were thoroughly mixed followed by incubation for 30 min at 50°C. Trichloroacetic acid (1%; 0.25 ml) was added to the mixture. A volume of 0.25 ml of the mixture was mixed with distilled water (0.25 ml) and 0.1% (w/v) ferric chloride (0.4 ml). The absorbance was recorded at 700 nm after 30 min. Increased absorbance is indicative of high reducing power. Gallic acid was used as standard.

#### Total antioxidant capacity (Phosphomolybdate assay)

The total antioxidant potency of test compounds was investigated by phosphomolybdate method of Umamaheswari and Chatterjee [26]. An aliquot of 0.1 ml of different concentrations (50, 100 and 200 μg/ml) of each sample was added to 1 ml of reagent (0.6 M H_2_SO_4_, 0.028 M sodium phosphate , 0.004 M ammonium molybdate) and incubated for 90 min at 95°C in a water bath. Absorbance was recorded at 765 nm after the samples cooled to room temperature. Ascorbic acid served as standard.

#### Acute toxicity studies in rat

For acute toxicity study, 42 male Sprague Dawley rats of good health were randomly divided into seven groups. Animals were off feed but have open access to water 15 h prior of test samples. Group I served as control group and received 15 % DMSO in olive oil intraperitoneally. However, Group II, III, IV, V, VI, and VII received 500, 400, 300, 200, 100 and 50 mg/kg of SCEE respectively in DMSO. General behavior of animals was noted after 120 min of treatment. Food and water were given *ad libitum*. Animals were screened for mortality and morbidity for 15 days [27].

### Experimental design for *in vivo* study

Male Sprague Dawley rats (180–200 g) of seven weeks old were used as animal model in this study. They were maintained in cages at room temperature of 25±3°C with a 12 h light/dark cycle and free access to water and feed. The study protocol was approved (No.0244) by the ethical committee of Quaid-i-Azam University, Islamabad, Pakistan for laboratory animal care and experimentation.

Patrick et al. [28] protocol with slight modification was followed to study the antioxidant potential of SCEE. Forty two male rats were randomly distributed into 7 groups (6 rats/group). Group I was remained untreated. Group II was treated with 15% DMSO in olive oil (1 ml/kg b.w) and have free access to food materials. Animals of Group III, IV, V and VI received 1 ml/kg b.w of CCl_4_ (20% in olive oil; v/v) intraperitoneally on alternative days for four weeks. Group III was treated with CCl_4_ only while group IV with 200 mg/kg b.w of silymarin in DMSO after CCl_4_ administration. Group V received 150 mg/kg b.w of SCEE and group VI received 300 mg/kg b.w of SCEE intragastrically, in DMSO after CCl_4_ treatments. Animals of group VII were given only SCEE in DMSO at dose of 300 mg/kg b.w intragastrically. After 24 h of the last treatment, all the animals were dissected. Blood was collected from heart by 3 ml syringe; the liver was removed and rinsed in ice-cold saline solution. Half liver was preserved in formaldehyde for histology and half was treated with liquid nitrogen and preserved at -20°C for further analysis.

### Liver marker enzymes assessment in serum

Liver marker enzymes in serum such as aspartate transaminase (AST), alanine transaminase (ALT), alkaline phosphatase (ALP), gamma glutamyltransferase (γ-GT) and lactate dehydrogenase (LDH) were analyzed by using standard AMP diagnostic kits (Stattogger Strasse 31b 8045 Graz, Austria).

### Assessment of antioxidant status

For antioxidant status assessment of different groups, 70 mg of liver tissue was homogenized in 10 volumes of 100 mM KH2PO4 buffer containing 1 mM EDTA (pH 7.4) and centrifuged at 12,000 × g for 30 min at 4°C. The supernatant was collected and used for determination of antioxidant enzymes and protein profile. The concentration of protein was estimated following the method of Lowry et al. [29] and antioxidant enzymes, including the activity of catalase (CAT), peroxidase assay (POD) [30], superoxide dismutase (SOD) [31], glutathione Stransferase assay (GST) [32], glutathione reductase (GSR) [33], glutathione peroxidase (GSHPx) [34], reduced glutathione assay (GSH) [35] and lipid per oxidation assay (TBARS) [36] were performed on hepatic samples.

### Liver histology

For histology small pieces of liver tissue from each group were fixed for 3–4 h in fixative sera followed by dehydration with ascending grades of alcohol (80%, 90%, and 100%) and transferred in cedar wood oil. When tissues become clear then all tissues were embedded in paraffin and prepared blocks for further microtomy. Thin sections 3–4 μm were prepared with microtome; wax was removed, stained with hemotoxylin-eosin and photographed under light microscope at 40×.

### Statistical analysis

All values are mean of triplicates. One way ANOVA analysis was carried by using Statistix 8.1 to assess the difference between various groups. The graph pad prism was used to calculate IC_50_ values. Correlation between IC_50_ values of different assays with total flavonoids and total phenolics was calculated by Pearson^’^s correlation coefficient with a significance level of P<0.05.

## Results and discussion

### Extract and fraction yield

**Figure 1:**
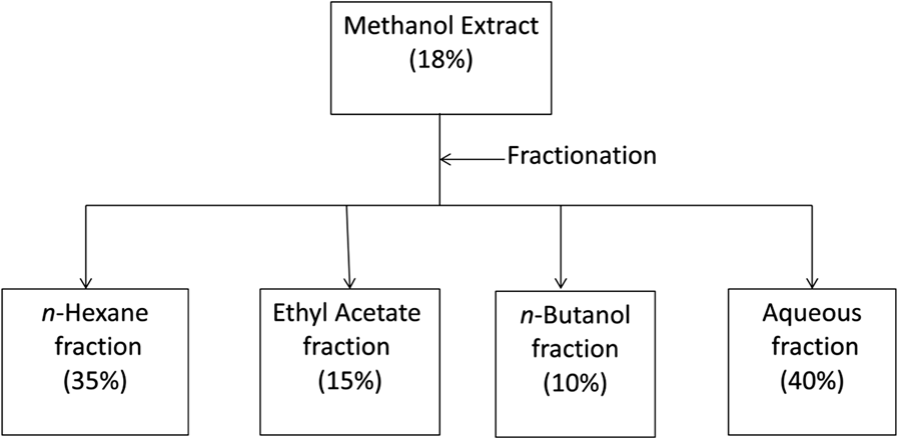
**Flow sheet of extraction and fractionation of *S. cordata *** **with percent yield.**

*S. cordata* crude methanol extract gave a yield of 18 percent (w/w), proceeded to further fractionation by using different organic solvents based on a difference of polarity index. Non polar *n*-hexane yield 35% fraction, while polar ethyl acetate, *n*-butanol yield 15% and 10% respectively. Residue soluble fraction known as aqueous fraction gave a yield of 40% (Figure 1). Mistry et al. [14] also reported extract yield in a similar range (17.6%) but solvent with different polarity and plant part.

### Phytochemical analysis

#### Total phenolic content and total flavonoid content

Flavonoids and phenolics are known to have good antioxidant capacity and it is likely that the antioxidant activity of extract/fraction might be due to these compounds. Therefore these are quantified to show its relation with antioxidant potential. Table 1 shows the quantity of total flavonoid and total phenolic content.

**Table 1:** **Quantitative profile of total flavonoids content and total phenolic content in the methanol extract and various fractions of *S. cordata***

**Extract/Fraction**	**Total flavonoid content (mg rutin equivalent/g extract/fraction)**	**Total phenolic content (mg gallic acid equivalent/g extract/fraction)**
SCME	154±6.7^a^	286.7±17.5^c^
SCHE	66±5.1^e^	49.0±3.6^d^
SCEE	312±1^b^	683.3±15.3^a^
SCBE	246±1^c^	485.3±13.2^b^
SCAE	88±5.6^d^	24.6±2.7^e^

Each values represented in tables are means ± SD (N=3). Values with different superscripts within same column are significantly different (P<0.05). (SCHE); *n*-hexane fraction, (SCEE); ethyl acetate fraction, (SCBE); *n*-butanol fraction, (SCAE), aqueous fraction.

Maximum quantity of total flavonoid content was observed in SCEE (312±1 mg rutin equivalent /g dry extract) while the lowest in SCHE (66±5.1 mg rutin equivalent/g dry extract). SCEE > SCME > SCBE > SCAE > SCHE descending order of total flavonoid content was observed. Maximum total phenolic content was recorded in SCEE (683.3±5.3 mg gallic acid equivalent/g dry extract) while the lowest quantity was observed in SCAE (24.6±2.7 mg gallic acid equivalent /g dry extract). Descending order of SCEE > SCBE > SCME > SCHE > SCAE was recorded for total phenolic content.

#### Thin layer chromatography

Thin layer chromatography (TLC) was performed by using 10 standards of flavonoids and phenolics. *S. cordata* methanol extract and all the derived fractions showed the presence of compounds by using stain. But only SCBE and SCEE fractions displayed the presence of some of the compounds with Rf values similar to that of the standards used as reference. Catechin, rutin and caffeic acid were observed in SCBE while apigenin in SCEE (data not shown).

Catechin is important polyphenolic compound and has shown many beneficial health associated effect in laboratory as well as in clinics due to their antioxidant and free radical scavenging activity along with stimulation of endogenous antioxidants. Catechin and its metabolites have shown potential as neuroprotective, antiapoptotic and anti-inflammatory in clinical disorders [37]. Caffeic acid is reported to have antimetastatic and antitumour activity by suppressing MMP-9 enzyme, an important actor of metastasis and cancer onset [38]. Rutin is important secondary metabolite in many plants and has been reported as hepatoprotective, antioxidant and anti-inflammatory agent [39]. Apigenin is known as a potent antioxidant and anti-inflammatory agent [40].

### Antioxidant assays

*In vitro* antioxidant test are very useful, time saving and economic activity to investigate the antioxidant potential of a plant extract and/or pure compound before commencing the extract/compound to the *in vivo* model for the antioxidant potential against free radicals. There are so many well established protocols for screening antioxidant potential against different types of free radicals. Here we elaborated anti free radical potential of *S. cordata* against different free radicals.

#### DPPH radical scavenging activity

DPPH possesses a proton free radical with property of absorption that decreases on exposure of proton radical scavengers. *S. cordata* methanol extract and various fractions showed good DPPH radical scavenging activity. DPPH radical scavenging activity was changed notably by various fractions. SCEE showed the lowest IC_50_ (135±1.2 μg/ml) against DPPH free radicals while SCHE showed highest IC_50_ value (795±5.3). IC_50_ values are given in Table 2. Results obtained in this study suggest that DPPH scavenging activity can be enhanced by the partition of crude methanol extract with ethyl acetate organic solvent.

**Table 2:** **IC**_**50 **_**values of different antioxidant assays of extract and various fractions of *S. cordata***

**Activity**	**IC**_**50**_**(μg/ml)**					
	**SCME**	**SCHE**	**SCEE**	**SCBE**	**SCAE**	**Standard**^**a**^
DPPH scavenging activity	235±1.4^c^	795±5.3^a^	135±1.2^e^	147±1.4^d^	275±3.2^b^	25±1.1^f^
Hydrogen peroxide scavenging activity	272±1.8^c^	469±2.7^b^	154±2.3^e^	223±2.1^d^	622±3.7^a^	99±2.2^f^
Hydroxyl radical scavenging activity	298±2.1^c^	578±4.2^a^	189±1.5^d^	206±1.3^e^	455±4.1^b^	32±0.9^f^
ABTS scavenging activity	259±0.9^a^	260±2.1^a^	143±0.8^d^	224±1.4^c^	236±2.2^b^	55±1.3^e^
Anti lipid per oxidation activity	212±3.1^c^	276±2.6^b^	119±1.0^e^	153±1.1^d^	314±2.8^a^	31±0.8^f^
β -carotene bleaching activity	221±1.7^c^	647±3.9^a^	153±1.1^d^	156±1.7^d^	249±1.6^b^	35±1.7^e^
Superoxide radical scavenging activity	139±2.0^c^	197±2.3^b^	114±1.3^d^	136±2.1^c^	242±3.1^a^	41±2.3^e^

Values are expressed as mean±SD (N=3), ^a^=Ascorbic acid. Means with superscripts with different letters in the rows are significantly (P<0.05) different from each other. (SCHE); *n*-hexane fraction, (SCEE); ethyl acetate fraction, (SCBE); *n*-butanol fraction, (SCAE), aqueous fraction.

#### Hydrogen peroxide radical

Hydrogen peroxide is the reactive oxygen metabolite causing damage to the cell at very low concentration of 10 μM. It is produced as a result of dismutation of superoxide radicals or directly or indirectly by some enzymes. Free solubility in aqueous, makes it freely movable across biological membranes. Deleterious effects include degradation of heme protein, inactivation of enzymes and oxidation of DNA, lipids, -SH groups and keto acid [41]. It reacts with Fe^2+^ and possibly Cu^2+^ ions to form hydroxyl radicals, which induce many toxic effects [42].

Hydrogen peroxide precursor of toxic hydroxyl radical is non reactive free radical species. Scavenging activity of extract and various fractions was observed to be concentration dependent (Additional file 1: Figure S1). SCEE illustrated the lowest IC_50_ (154±2.3 μg/ml) while other fractions showed activity in the order of SCBE < SCME < SCHE < SCAE with IC_50_ of 223±2.1, 272±1.8, 469±2.7 and 622±3.7 μg/ml, respectively (Table 2).

#### Hydroxyl radical scavenging activity

Hydroxyl is very toxic free radical, causing damage to deoxyribonucleic acids and proteins. In the present experiment, the evidence of •OH scavenging activity by *S. cordata* extract and fractions were obtained through the deoxyribose system. *S. cordata* samples scavenged •OH radicals in a concentration dependent manner and organic solvent fraction type wise. Best hydroxyl radical scavenging activity was shown by SCEE with IC_50_ value of 189±1.5 μg/ml while the highest value range was recorded by SCHE (578±4.2 μg/ml) as shown in Table 2. The strong antioxidant activity of SCEE can be utilized as a source of natural antioxidant in oxidative stress for minimizing the detrimental effects of hydroxyl radical in the body.

#### ABTS radical scavenging activity

ABTS radical scavenging activity of extract and various fractions was evaluated by using 2, 2 azobis-(3-ethylbenzothiozoline-6-sμlphonic acid). Best ABTS radical scavenging effect was shown by SCEE (IC_50_ =143±0. 8 μg/ml), higher than the standard used but comparable. IC_50_ values of SCME versus SCHE and SCBE versus SCAE displayed no significant difference (Table 2). ABTS radical scavenging activity was observed to be dose dependent.

#### Anti lipid peroxidation potential

Egg yolk lipids on reaction with ferrous sulphate undergo rapid non enzymatic peroxidation. Lipid peroxides are possible causative agent of inflammation, oxidative stress, metabolic malfunctioning and aging. The IC_50_ values of lipid peroxidation activity of *S. cordata* extract and various fractions is summarized in Table 2. Maximum anti-lipid peroxidation activity was observed by SCEE and lowest by SCAE with IC_50_ values of 119±1.0 μg/ml and 314±2.8 μg/ml respectively.

#### Beta carotene scavenging activity

Antioxidant potential of *S. cordata* extract and fraction was determined by β-carotene bleaching method based on the oxidation of linoleic acid. Linoleic acid hydroperoxides react with β-carotene molecule resulting in the rapid disappearance of color. The presence of antioxidant can obstruct the extent of β-carotene by acting on linoleate free radicals and other free radicals formed in the system. So the absorbance rapidly decreased in samples without antioxidants whereas in the presence of an antioxidant, they maintained their absorbance and color for a longer period. Among *S. cordata* samples, SCEE showed promising (IC_50_=153±1.1 μg/ml) β-carotene bleaching antioxidant activity followed by SCBE (156±1.7 μg/ml) > SCME (221±1.7 μg/ml) > SCAE (249±1.6 μg/ml) > SCHE (647±3.9 μg/ml) (Table 2). This activity observed to be concentration dependent.

#### Superoxide radical scavenging activity

Oxidation is an important phenomenon of life, but apart from so many crucial processes of life, during normal metabolism of oxygen, various free as well as superoxide’s are continuously produced. It is considered a weak oxidant, but gives rise to toxic and powerful oxidant such as hydroxyl radical and singlet oxygen resulting in many diseases [43].

*S. cordata* showed superoxide radical scavenging activity with best IC_50_ of 114±1.3 μg/ml for SCEE, while remaining extract and fractions with IC_50_ of 136±2.1, 139±2.0, 197±2.3, 242±3.1 μg/ml for SCBE, SCME, SCHE, SCAE respectively (Table 2).

#### Reducing power

Reducing power of the plant was determined by using potassium ferricyanide reduction method. Now it is an established phenomenon that reducing power is linked with antioxidant potential and it correlates with phenolic constituents in several vegetables. In reducing power assay, the oxidation form of iron (Fe^+3^) in ferric chloride is converted to ferrous (Fe^+2^) by antioxidant compounds. Extract and different fractions of *S. cordata* expressed good reducing power activity. SCEE and SCME showed reducing power with the approximate absorbance value 0.647±0.08 and 0.583± 0.07 respectively at maximum dose of 200 μg/ml in comparison to standard gallic acid (1.13±0.03).

Now it is an established phenomenon that reducing power is linked with antioxidant potential and it correlates with phenolic constituents in several vegetables/foods. The reducing power of a compound may serve as an important marker of its possible antioxidant activity. However, the activities of antioxidants have been ascribed to various mechanisms such as prevention of chain initiation, decomposition of peroxides, reducing capacity and radical scavenging [44].

#### Phosphomolybdenum assay

Phosphomolybdenum assay principal follows the chemistry of conversion of Mo (VI) to Mo (V) by compound/extract having antioxidant property and resulting in formation of green phosphate/Mo (V) compound with maximum absorption at 695 nm. In the present assay all the *Sida cordata* different samples showed good total antioxidant index in order of SCEE > SCME > SCBE > SCAE > SCHE at a highest dose of 200 μg/ml. SCEE value of antioxidant index (1.129±0.01) is comparable with standard ascorbic acid (1.73±0.23).

### Correlation between IC_50_ values and phytochemical constituents

Correlation between IC_50_ values of various radical scavenging activities with total phenolic and total flavonoids constituents is given in Table 3. All the fractions showed good correlation with total flavonoid contents (R^2^=0.57 - 0.92) and total phenolic contents (R^2^=0.44 - 0.97). Significant correlations (P<0.01, P<0.05) of IC_50_ were observed with total flavonoid contents and total phenolic contents in all antioxidant assays except DPPH scavenging activity and β-carotene bleaching activity.

**Table 3:** **IC**_**50 **_**correlation with total flavonoid and total phenolic contents**

**Activity**	**Correlation R**^**2**^	
	**Flavonoids**	**Phenolics**
DPPH scavenging activity	0.57	0.46
Hydrogen peroxide scavenging activity	0.78*	0.86*
Hydroxyl radical scavenging activity	0.89**	0.85*
ABTS scavenging activity	0.67*	0.65
Anti lipid per oxidation activity	0.92**	0.97**
β - carotene bleaching scavenging activity	0.55	0.44
Superoxide radical scavenging activity	0.73*	0.82*

Values are expressed as mean±SD (N=3). Values within the same column with different superscripts letter are significantly different. *=P<0.05; **=P<0.01.

### *In vivo* studies

Besides *in vitro* antioxidant activity, *in vivo* study was performed on SCEE, showing best antioxidant potential as well as good flavonoid and phenolic contents among *S. cordata* methanol extract and its various derived fractions during *in vitro* assays. Flavonoids are considered good hepatoprotective agents [45]. To assess this potential of SCEE fraction, it was applied against CCl_4_ induced hepatic toxicity. CCl_4_ is extensively used in animal models to look into chemical toxin induced hepatic damage. It induces cirrhosis, necrosis and fats deposition resulting from formation of trichloromethyl free radicals [46].

For *in vivo* study, doses of 150 and 300 mg/kg b.w were selected for hepatoprotection against CCl_4_-induced toxicity study.

CCl_4_ was injected on alternate day (thirty days) to induce hepatotoxicity in animal model. CCl_4_ is recognized to cause liver damage marked in striking augmentation in serum profile of LDH, bilirubin, γ-GT, ALP and aminotransferase enzymes (ALT and AST), especially ALT, which is considered the specific and primary indicator of hepatic injury. Accordingly, our results also demonstrated a significant increase in the profile of ALT, AST and LDH after CCl_4_ treatment (Table 4), hence confirming the liver damage at the cellular level in CCl_4_-treated rats. SCEE treatment markedly lowered the level of marker enzymes near to that of control groups.

**Table 4:** Effect of SCEE treatment on different liver markers

**Group**	**LDH (U/L)**	**Billirubin (mg/ml)**	**ALT (U/L)**	**AST (U/L)**	**ALP (U/L)**	**γ –GT (U/L)**
Control	1847±21.2^e^	0.33±0.1^b^	22.67±1.5^b^	38.7±3.5^d^	56.0±1.0^cd^	0.937±0.0^de^
Oil+DMSO	1964±30.2^cd^	0.33±0.1^b^	21±1.00^bc^	40.3±1.5^cd^	54.0±1.0^d^	0.863±0.0^e^
CCl_4_ (1 ml/kg)	3268±55.7^a^	0.90±1.0^a^	43.3±1.5^a^	70.0±3.0^a^	91.3±2.5^a^	2.58±0.21^a^
Silymarin (200 mg/kg)+CCl_4_	1753±15.3^f^	0.37±0.1^b^	18.0±1.0^a^	44.7±2.5^cd^	60.3±2.5^c^	1.16±0.10^cd^
SCEE (150 mg/kg)+CCl_4_	2207±21.4^b^	0.43±0.1^b^	20.0±1.0^bc^	56.3±3.1^b^	71.3±1.5^b^	1.77±0.20^b^
SCEE (300 mg/kg)+CCl_4_	2054±48.0^c^	0.43±0.1^b^	20.5±0.5^bc^	47.0±2.6^c^	69.0±1.0^b^	1.35±0.17^c^
SCEE (300 mg/kg)	1902±20.4^d^	0.33±0.1^b^	22.0±1.0^b^	40.7±2.5^cd^	58.7±1.5^cd^	1.04±0.10^de^

Values are expressed as mean±SD (N=3). Values within the same column with different superscripts letters are significantly (P<0.05) different. (SCHE); *n*-hexane fraction, (SCEE); ethyl acetate fraction, (SCBE); *n*-butanol fraction, (SCAE), aqueous fraction.

In the present experiments after the treatment of CCl_4_ the levels of the serum markers got elevated, suggesting liver injuries in CCl_4_ treated groups. A similar profile was also observed by Khan and colleagues [47] in rat after treatment with CCl_4._

The effect of SCEE on different antioxidant enzymes (CAT, POD, SOD, GST, GSH-P_X_ and GSR) profile is given in Table 5. Administration of CCl_4_ markedly (P < 0.05) reduced the level of antioxidant enzymes in the CCl_4_ treated group when compared to that of control group, but its profile was obviously reversed by SCEE dose dependently.

**Table 5:** Effects of SCEE treatment on antioxidant enzymes profile of liver

**Group**	**CAT (U/min)**	**POD (U/min)**	**SOD(U/mg protein)**	**GST (nM/min/mg protein)**	**GSH-Px (nM/min/mg protein)**	**GSR (nM/min/mg protein)**
Control	6.1±0.2^ab^	10.1±0.2^a^	4.1±0.2^a^	129±9.5^a^	81.1±4.8^a^	131.3±8.5^ab^
Oil+DMSO	5.7±0.2^bc^	9.9±0.2^a^	3.9±0.1^a^	113±6.0^bc^	73.7±8.0^ab^	121.3±8.5^ab^
CCl_4_ (1 ml/kg)	1.8±0.1^e^	4.3±0.2^b^	1.2±0.2^d^	56±8.0^e^	47.0±9.2^c^	65.7±6.1^d^
Silymarin (200 mg/kg)+CCl_4_	5.4±0.1^c^	8.4±0.3^b^	3.2±0.1^b^	107±8.0^bc^	73.3±5.5^ab^	111±6.0^bc^
SCEE (150 mg/kg)+CCl_4_	3.9±0.2^d^	6.6±0.4^c^	2.3±0.2^c^	82.7±5.5^d^	60.3±3.1^bc^	94.3±6.1^c^
SCEE (300 mg/kg)+CCl_4_	4.4±0.3^d^	7.3±0.5^c^	2.6±0.2_c_	101±6.0^cd^	65.7±2.9^ab^	111±5.3^bc^
SCEE (300 mg/kg)	6.3±0.2^a^	10.2±0.3^a^	4.1±0.1^a^	123±9.7^ab^	71.7±3.5^ab^	135.7±11.4^a^

Values are expressed as mean±SD (N=3). Values within the same column with different superscripts letters are significantly (P<0.05) different. (SCHE); *n*-hexane fraction, (SCEE); ethyl acetate fraction, (SCBE); *n*-butanol fraction, (SCAE), aqueous fraction.

Antioxidant enzymes represent protective system against tissue damage by oxidation. SOD transformed O_2_ into H_2_O_2_. GSH-Px and CAT metabolize H_2_O_2_ to non damaging products. GSH antioxidant network play elementary role in cellular protection against free radical species. This system comprises GSH and a group of functionally connected enzymes of which GSR is accountable for GSH regeneration while GSH-Px and GST function jointly with GSH for conversion of H_2_O_2_ to hydroperoxides [48].

GSH-Px that play a key role in the free radical neutralization was significantly lowered after CCl_4_ treatments but restored in rats treated with SCEE. These findings suggest that CCl_4_ probably through its converted forms in the liver after administration, hinder the activity of GSH-Px. Similarly, after CCl_4_ treatment levels of CAT, POD, and SOD were also decreased and normalized with the treatment of SCEE.

P450-2E1 convert CCl_4_ to CCl_3_ in the animal tissue which induce toxicity in tissues but in the same time GSH detoxifying pathway also activated, resulting in the conjugation of toxic metabolite CCl_3_[17]. Recknagel et al. [49] in their study of CCl_4_ toxicity induction in liver showed that GSH is a key player in removing toxic metabolite of CCl_4_, and CCl_4_ toxicity starting to appear when GSH profile is exhausted.

The effect of SCEE on protein, TBARS, and GSH profile is displayed in Table 6. Protein and GSH levels were significantly reduced due to the toxicity of CCl_4_. SCEE treatment dose dependently protected its alteration and showed profile near to control group. TBARS levels were augmented significantly and reversed by SCEE treatment.

**Table 6:** Effects of SCEE administration on protein, TBARS and GSH profile of liver

**Group**	**Protein (μg/mg tissue)**	**TBARS (nM/min/mg protein)**	**GSH (μM/g tissue)**
Control	3.1±0.1^a^	1.3±0.2^d^	29.3±2.5^a^
Oil+DMSO	2.6±0.2^b^	1.4±0.2^d^	25.7±2.1^a^
CCl_4_ (1 ml/kg)	1.3±0.2^d^	3.7±0.4^a^	11.3±2.5^c^
Silymarin (200 mg/kg)+CCl_4_	2.8±0.2^ab^	1.8±0.2^cd^	27.3±3.5^a^
SCEE (150 mg/kg)+CCl_4_	1.5±0.1^cd^	2.7±0.1^b^	17.7±3.1^bc^
SCEE (300 mg/kg)+CCl_4_	1.8±0.1^c^	2.3±0.2^bc^	24.0±2.7^ab^
SCEE (300 mg/kg)	2.8±0.1^ab^	1.6±0.2^cd^	31.0±2.7^a^

Values are expressed as mean±SD (N=3). Values within the same column with different superscripts letters are significantly (P<0.05) different. (SCHE); *n*-hexane fraction, (SCEE); ethyl acetate fraction, (SCBE); *n*-butanol fraction, (SCAE), aqueous fraction.

SCEE administration also prevented the CCl_4_ induced increase in liver TBARS levels, suggesting that SCEE obstruct lipid peroxidation and its promulgation reactions as expressed by *in vitro* assays. CCl_4_ caused noticeable toxicity by increasing the hepatic lipid peroxides, as marked by higher levels of hepatic TBARS. It is well known that CCl_4_ induce liver toxicity is attributed to the reductive dehalogenation of CCl_4_, catalyzed by CYP450 in the hepatic endoplasmic reticulum, leading to the generation of trichloromethyl peroxy radicals (•CCl_3_), which is reported to be an unstable complex and belongs to highly reactive species [50]. This free radical reacts with lipids of membrane, leading to per oxidation, and may also cause cell damage by covalently binding to proteins and lipids resulting in harmful processes. SCEE may give cell protection by hindering CCl_4_-mediated lipid peroxidation, hence blocking the generation of free radical derivatives [49]. If the elevated TBARS levels are taken into consideration, CCl_4_ exposure increased lipid peroxidation, it also lowered intracellular GSH profile, demonstrating that GSH depletion might arise from the detoxification of CCl_4_ by GSH conjugation. Therefore, these combined results strongly suggest that SCEE also acts as an antioxidant in animal model. This effect could be characteristic of the antioxidant activity of the *S. cordata* SCEE fraction used, which markedly diminished the oxidative hazard and paved to reinstatement of normal physiological features. In addition, the antioxidant enzymes in rats co-treated with SCEE have activities similar to those of controls. These effects can be attributed here to SCEE by playing a role during the early stages in CCl_4_-induced hepatic damage, diminishing lipid peroxidation consequently and improving cellular antioxidant position, thereby obstructing AST, ALT and LDH outflow from the liver.

**Figure 2:**
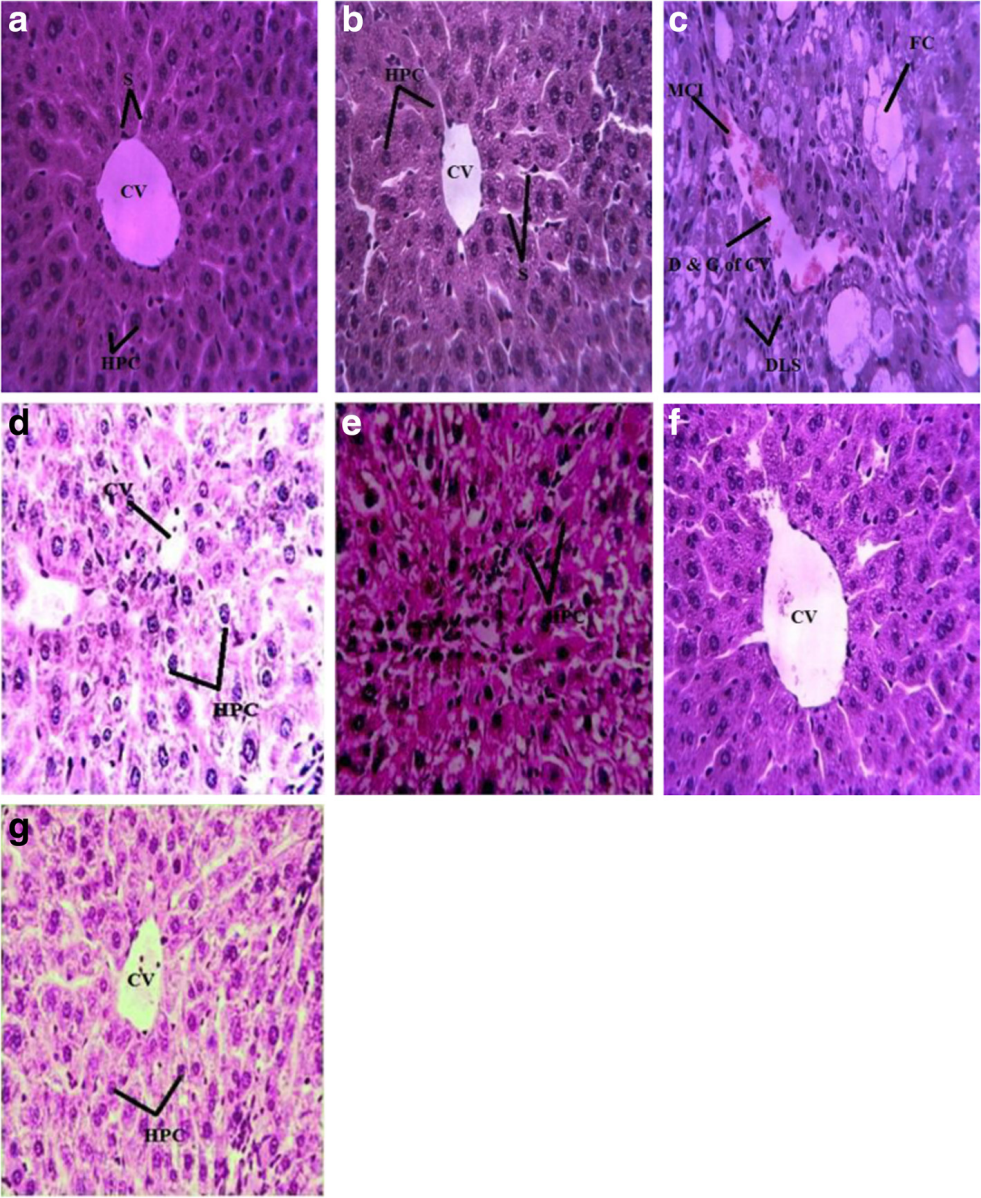
**Rat liver microscopic photograph (H & E stain).2a**; Represents normal liver section, **2b**; Vehicle control, **2c**; CCl_4_ treated control, **2d**; CCl_4_+silymerin treated, **2e**; CCl_4_ +SCEE 150 mg/kg treated, **2f**; CCl_4_+SCEE 300 mg/kg treated **2g**; only SCEE 300 mg/kg treated. CV (Central vein), S (Sinusoids) and HPC (Hepatocytes). DLS (Degeneration of lobular shape), FC (Fatty change), and D& C (Dilation and congestion of central vein).

Liver tissue slides were stained with hemotoxylin and eosin (Figure 2). CCl_4_ administration as depicted in Figure 2c shows central vein dilation, inflammatory cells infiltration, cellular hypertrophy, necrosis and degeneration of lobular architecture. Similar alterations were reported by Khan et al. [51] in their study in rat after CCl_4_ treatment. SCEE administration resulted in minimizing of such morphological alterations dose dependently and clear difference can be observed in histoarchitecture to that of CCl_4_ treated. Animals treated with SCEE alone did not revealed any alteration in morphology of the liver. However, only macrosteatosis in hepatocytes was reported by Mistry et al. [14] with an ethanol extract of *S. cordata* leaves. This difference in alteration of histoarchitecture might be due to the different animal breed and/or intensity and duration of the CCl_4_ treatment.

The main flavonoid present in the SCEE, identified by TLC fingerprinting was apigenin. Apigenin is known to have a role in the amplification of profile of antioxidant enzymes i.e. superoxide dismutase and erythrocyte glutathione reductase [52]. It induces reduction of plasma profile of low density lipoprotein, inhibition of platelet aggregation and reduction of cell proliferation [53]. Jeyabal et al. [54] have shown that apigenin give protection to liver in term of oxidative stress and DNA damage against N-nitroso-diethylamine induced and phenobarbitol promoted liver carcinogenesis in rats when fed at a dose of 25 mg/kg body weight. We suppose here that apigenin alone or combination with other unknown components may have a role in the reduction of hepatotoxicity induced by CCl_4_ in rat model. Based on the experimental results in the present study SCEE may play a key role in therapeutics by free radical capturing and activation of antioxidant enzymes may lead to the protection of the liver against CCl_4_ induced injury. But complete study is required to verify the mechanism of protection against CCl_4_ by SCEE at the molecular level.

## Conclusion

Results obtained in the present study shows that SCEE is an active herbal protective drug against hepatotoxicity, but complete investigation is required to isolate the hepatoprotective compound in pure form for drug development on a large scale.

## Competing interest

The authors declare that they have no competing interests.

## Authors’ contributions

NAS made significant contributions to conception, acquisition of data, analysis, drafting of the manuscript. MRK has made substantial contribution to and design, interpretation of data, drafting and revising the manuscript for intellectual content. BA, FN, UR and RAK (Khan RA 0000-0003-0453-2090) participated in the design and collection of data and analysis. All authors read and approved the final manuscript.

## Pre-publication history

The pre-publication history for this paper can be accessed here:

http://www.biomedcentral.com/1472-6882/13/276/prepub

## Supplementary Material

Additional file 1: Figure S1 Antioxidant activity of S. cordata methanol extract and its various derived fractions at different concentrations. (a) DPPH radical scavenging activity (b) Hydrogen peroxide scavenging activity (c) Hydroxyl radical scavenging activity (d) ABTS radical scavenging activity (e) Anti lipid peroxidation activity (f) Beta carotene activity (g) Superoxide radical scavenging activity (h) Reducing power potential (i) Total antioxidant potential. Click here for file
