# Supplementary material for: Investigation on flavonoid composition and anti free radical potential of Sida cordata
Source: BMC Complement Altern Med. 2013 Oct 22;13:276. doi: 10.1186/1472-6882-13-276 (PMC3874743; doi:10.1186/1472-6882-13-276)
Supplement: Additional file 1: Figure S1 — Antioxidant activity of S. cordata methanol extract and its various derived fractions at different concentrations. (a) DPPH radical scavenging activity (b) Hydrogen peroxide scavenging activity (c) Hydroxyl radical scavenging activity (d) ABTS radical scavenging activity (e) Anti lipid peroxidation activity (f) Beta carotene activity (g) Superoxide radical scavenging activity (h) Reducing power potential (i) Total antioxidant potential. [file 1472-6882-13-276-S1.docx]

**Figure 3a**.

**Figure 3b**.

**Figure 3c**.

**Figure 3d**.

**Figure 3e**.

**Figure 3f**.

**Figure 3g**.

**Figure 3h**.

**Figure 3i**

**Figure** .Antioxidant activity *S. cordata* methanol extract and it,s various derived fractions at different concentrations of 50,100 and 200 ug/ml **(a)**DPPH radical scavenging activity**(b)**Hydrogen peroxide radical scavenging activity**(c)**Hydroxykl radical scavenging activity**(d)** ABTS radical scavenging activity**(e)** Anti lipid peroxidation activity**(f)** Beta carotene activity**(g)** Superoxide radical scavenging activity**(h)** Reducing power potential**(i)** Total antioxidant activity potential. Each value is expressed as mean±SD (N=3)

Figure. TLC plates showing different compounds in *S. cordata* methanol extract and its derived various fractions. Bands encircled without tagging are unknown compounds (not present in standards used). 1, 2,3,4,5 represent SCEE, SCAE, SCHE, SCME, and SCBE respectively.
